# A Waist-Mounted Interface for Mobile Viewpoint-Height Transformation Affecting Spatial Perception

**DOI:** 10.3390/s26020372

**Published:** 2026-01-06

**Authors:** Jun Aoki, Hideki Kadone, Kenji Suzuki

**Affiliations:** 1 Empowerment Informatics Program, University of Tsukuba, Tsukuba 3058573, Japan; 2 Department of Cybernics Medicine, Institute of Medicine, University of Tsukuba, Tsukuba 3058575, Japan; 3 Institute of Systems and Information Engineering, University of Tsukuba, Tsukuba 3058573, Japan

**Keywords:** human augmentation, viewpoint-height transformation, spatial perception, augmented reality, human–computer interaction

## Abstract

Visual information shapes spatial perception and body representation in human augmentation. However, the perceptual consequences of viewpoint-height changes produced by sensor–display geometry are not well understood. To address this gap, we developed an interface that maps a waist-mounted stereo fisheye camera to an eye-level viewpoint on a head-mounted display in real time. Geometric and timing calibration kept latency low enough to preserve a sense of agency and enable stable untethered walking. In a within-subject study comparing head- and waist-level viewpoints, participants approached adjustable gaps, rated passability confidence (1–7), and attempted passage when confident. We also recorded walking speed and assessed post-task body representation using a questionnaire. High gaps were judged passable and low gaps were not, irrespective of viewpoint. At the middle gap, confidence decreased with a head-level viewpoint and increased with a waist-level viewpoint, and walking speed decreased when a waist-level viewpoint was combined with a chest-height gap, consistent with added caution near the decision boundary. Body image reports most often indicated a lowered head position relative to the torso, consistent with visually driven rescaling rather than morphological change. These findings show that a waist-mounted interface for mobile viewpoint-height transformation can reliably shift spatial perception.

## 1. Introduction

Human augmentation aims to extend or support human perception and action in everyday contexts, and it is most effective when devices couple tightly to the user’s body and environment. This perspective follows broader accounts of embodiment and self-organization, where sensorimotor couplings can reorganize perception and action [1], and recent surveys outline how augmentations—ranging from wearables to extended reality (XR) interfaces—shift what actions feel possible [2]. Scoping reviews on the design for wearability further emphasize that attachment stability, comfort, and unobtrusive integration into everyday routines are critical but still under-specified targets for wearable devices [3]. Concrete domains such as exoskeletons, orthoses, and advanced prostheses make these links explicit by showing how design choices alter control policies and embodiment reports [4,5].

A large body of work shows that multisensory consistency can shift the felt boundaries of the body: the rubber hand illusion and full-body variants demonstrate that synchronized visuotactile cues induce ownership and alter perceived self-location [6,7,8]. Psychometric and theoretical accounts organize embodiment into ownership, agency, and self-location [9,10]. Beyond these classics, interactive and added limb systems reveal plasticity in body representation and control, including homuncular flexibility and changes in neural/body maps with extra robotic hands and arms [11,12,13,14,15], and prosthetic feedback has shown that illusory movement can improve control and modulate embodiment [16,17].

In addition to multisensory ownership, embodiment can also be shaped by purely visual scaling cues that relate the body to the environment—most notably eye height, which provides a strong metric for judging size and distance. Among sensory routes to augmentation, vision is especially potent because viewpoint height acts as a scaling cue that links the body to the world. Manipulating apparent body size or viewpoint height shifts perceived scale and size judgments in otherwise unchanged environments [18,19], and child-like or height-transforming embodiments in virtual reality (VR) can modulate object size perception, social evaluation, and affect [20,21,22]. For example, in immersive VR, conflicts between visually specified and posturally specified eye height produce predictable biases in egocentric distance estimates [23], and simulated eye-height manipulations can rescale perceived object size [24]. Similarly, body-size illusions causally rescale perceived size and distance in otherwise unchanged scenes [18]. Avatar and perspective choices also influence body part and self-localization and interpersonal behavior [25,26,27], with third-person viewpoints yielding distinct perceptual and attitudinal effects from first-person settings [28,29].

Moving from avatars to the real world, several systems have altered viewpoint height during natural locomotion. BigRobot elevated the first-person view to evoke a “giant” experience, whereas CHILDHOOD lowered it to a child-like vantage; both studies documented changes in subjective experience and interpersonal distance [30,31]. Related telexistence-style platforms combine first-person video with externalized or collaborative viewpoints for copresent tasks [32,33], and head-worn multisensory augmentation can further tune spatial awareness [34]. Low, predictable latency remains critical to preserve ownership and agency [35].

Ecological accounts make a specific prediction about scaling: passability judgments depend on the eye height ratio, so shifting eye height shifts the judged critical barrier height while the ratio remains roughly constant [36]. Locomotion work likewise shows that strategies for negotiating narrow gaps scale with body dimensions (e.g., shoulder width), including the onset of shoulder rotation near gaps [37,38]. Gap-affordance judgments in immersive virtual environments have also been examined across development (children, teens, and adults), highlighting age-related differences in conservative decision criteria under risk [39]. These ideas suggest that changing viewpoint height alone could update the decision criteria for gap passability, even when physical body size and kinematics remain unchanged.

Building on this background and our prior prototype exploring viewpoint transformation during walking [40], we target a visual route to augmentation—manipulating viewpoint height with a waist-mounted stereo camera to test whether scaling cues alone can update gap passability judgments, walking speed, and body image reports in real-world tasks.

**Figure 1:**
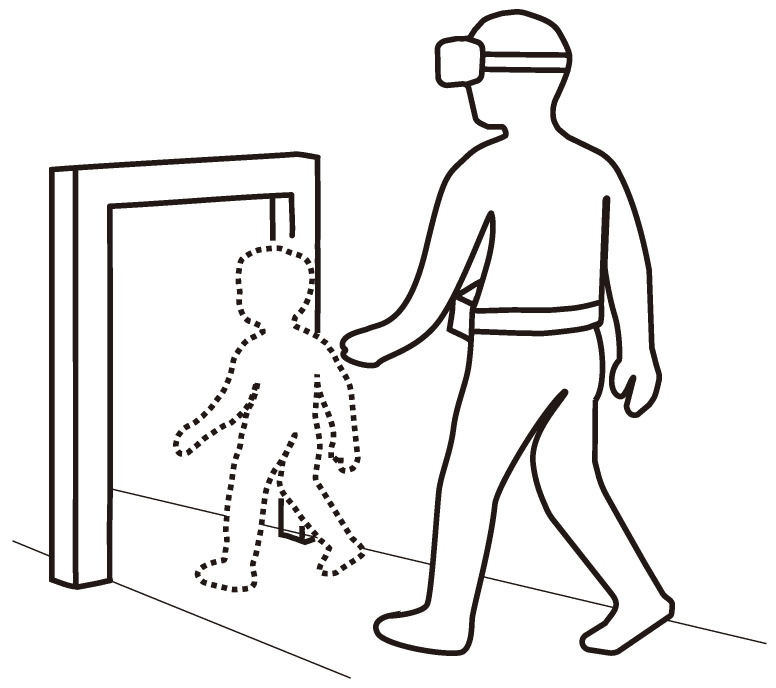
Overview of passing through the gap with viewpoint transformation.

Beyond basic science, altering perceived viewpoint height may support training and accessibility. Lowering the vantage can let caregivers and parents experience child-level spatial scale, while wheelchair level viewpoints can help clinicians and designers assess how easily spaces can be passed through and communicate body–environment scaling. These considerations motivate testing how viewpoint height alone reshapes gap passability judgment, body image, and walking speed reports during real-world walking (Figure 1).

### 1.1. Research Questions

We articulate three research questions concerning passability judgments, body representation, and walking speeds:

RQ1: To what extent does the perceived viewpoint height influence judgments about the passability of gaps at different heights?
RQ2: In what ways does the perceived viewpoint height shape subjective body representation and spatial-awareness reports?
RQ3: How does the perceived viewpoint height relate to walking speed near gaps of different heights?

### 1.2. Contributions

We present novel sensors and wearable technology for augmented reality (AR) simulation. Specifically, we propose a waist-mounted interface for mobile viewpoint-height transformation that supports untethered, natural walking while remapping a waist-mounted fisheye stereo view to a smartphone-shelled HMD with low latency. Stability is achieved by projecting the fisheye image onto a hemispherical mesh in Three.js, regularizing peripheral distortion, and maintaining a coherent virtual height view without desktop-class rendering. Even with standard (non-high-end) resolution and field of view (FOV), a simple interface with computational demands low enough to be handled by a portable computer altered spatial perception during real walking, indicating that high-grade optics and full-room tracking are not required.

### 1.3. Terminology

We use “body image” for conscious, reportable representations of one’s body size and appearance; “body schema” for action-oriented, largely implicit sensorimotor representations used for walking or moving; and “body representation” as an umbrella term covering both. To avoid ambiguity, we reserve body image for self-reports and body schema for behavioral indices derived from action.

## 2. Methods

**Figure 2:**
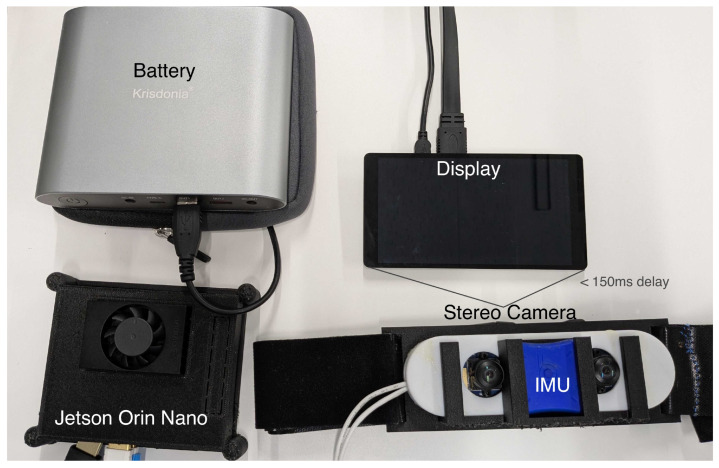
System Overview.

Prior work achieved viewpoint transformation with pan–tilt mechanisms [40], but such systems suffer from control delay, backlash, overshoot, jitter, cable load, and fragility, limiting prolonged use during walking. We therefore set three design goals and developed a system, as shown in Figure 2:

1. No moving parts: stable and robust operation during prolonged walking.
2. End-to-end delay <150 ms: preserves body ownership and presence [35].
3. Backpack integration: onboard computing, power, and sensors for rapid setup and mobility.

### 2.1. Visual Presentation

A waist-mounted stereo rig with two USB 3.1 fisheye cameras captures left/right views, and a six-inch display in smartphone-style VR goggles (VRG-S01BK, ELECOM) presents the images. Onboard processing runs on an NVIDIA Jetson Orin Nano Super housed in a backpack with a battery and wiring. To present the live streams in a web browser, we launch a lightweight local Python HTTP server on the Jetson to serve the browser-based rendering application; the browser (Google Chrome) acquires the stereo camera streams via getUserMedia and uses them as VideoTexture inputs. Stereo frames are rendered in real time using the Three.js 3D library (r128): each eye stream is applied as a video texture and mapped to the interior of a hemispherical surface.

Two PerspectiveCameras are used to render the left/right views to separate viewports in a side-by-side (SBS) format. The renderer uses a vertical field of view of $60^{\circ }$ with near/far planes of 0.1/100, and each eye stream is presented on an inward-facing hemispherical mesh implemented with SphereGeometry (radius $R_{\mathrm{mesh}}=30$ in scene units, 64×64 segments) with a fixed pre-rotation to align the optical axis (defined as the +Z direction in Three.js in the pre-rotated coordinate frame). To reduce latency, we disable anti-aliasing, fix the pixel ratio to 1.0, and use a VideoTexture with mipmaps disabled and linear filtering. Because stereo disparity is already contained in the captured left/right fisheye images, the two virtual cameras share the same pose and are used solely for SBS viewport rendering.

For the fisheye-to-hemisphere warp, we adopt an equidistant approximation (r∝θ). Let (θ,ϕ) denote the polar and azimuth angles of a vertex on the unit hemisphere, and let $\left(c_{x},c_{y}\right)$ be the center of the circular fisheye image with usable radius $r_{\max}$ (all in pixel units). In practice, we compute (θ,ϕ) from the unit direction d=(x,y,z) expressed in the pre-rotated mesh coordinate frame (i.e., after applying the same fixed pre-rotation used for the hemispherical mesh) as θ=arccos(z) and ϕ=atan2(y,x) (angles in radians). We compute the corresponding fisheye sampling coordinate as

(1) $$\rho =k\;\theta ,\;k=\frac{r_{\max}}{\theta_{\max}},\;u=c_{x}+\rho \cos\phi ,\;v=c_{y}+\rho \sin\phi ,$$

where $\theta_{\max}=\pi /2$ for a hemisphere. The fisheye texture is sampled at (u,v) (normalized to (u/W,v/H) in the implementation) and mapped onto the hemispherical surface; pixels outside the usable circle appear black in our camera output, yielding the same effect as masking.

Because fisheye projection models mainly diverge in the periphery, perceptual inconsistencies are expected to be smaller near the optical axis. We use the same fixed warp across all conditions; thus, any residual distortion remains constant within-subject and is unlikely to confound the viewpoint-height transformation.

### 2.2. Head Rotation

Two nine-axis inertial measurement units (IMUs; LPMS-B2) are attached to the head-mounted display (HMD) and the waist. Posture data are streamed over Robot Operating System 2 (ROS 2) and received in the browser via roslibjs. To preserve natural head scanning while suppressing whole-body turns during locomotion, we use the head IMU roll and pitch directly and correct only yaw by subtracting the waist yaw. This decoupling stabilizes the rendered heading and helps attribute downstream perceptual effects primarily to viewpoint height rather than to body rotation. This rotation compensation was enabled only in the Waist condition, where the camera was mounted on the waist and thus did not physically follow head turns. In the Head condition, because the camera was mounted on the HMD, head turns were inherently reflected in the captured video; therefore, no additional IMU-based camera rotation was applied.

Let $\left(\alpha_{h}\left(t\right),\beta_{h}\left(t\right),\psi_{h}\left(t\right)\right)$ denote the head IMU roll, pitch, and yaw, and let $\psi_{w}\left(t\right)$ denote the waist IMU yaw. We remove whole-body turns by using only the relative yaw

(2) $$\Delta \psi \left(t\right)=\mathrm{wrap}\;\left(\psi_{h}\left(t\right)-\psi_{w}\left(t\right)\right),$$

where wrap(·) maps angles to (−π,π].

In our implementation, a ROS node publishes a geometry_msgs/Vector3 message on /processed containing the angles (in radians) used by the Three.js camera: $m_{x}\left(t\right)=\Delta \psi \left(t\right)$ (yaw), $m_{y}\left(t\right)=\alpha_{h}\left(t\right)$ (roll), and $m_{z}\left(t\right)=\beta_{h}\left(t\right)$ (pitch). Following the Three.js XYZ Euler convention, we set

(3) $$\alpha \left(t\right)=m_{y}\left(t\right),\;\beta \left(t\right)=m_{z}\left(t\right),\;\gamma \left(t\right)=m_{x}\left(t\right),$$

and update the virtual camera orientation as

(4) $$R_{\mathrm{cam}}\left(t\right)=R_{x}\;\left(\alpha (t)\right)\;R_{y}\;\left(\beta (t)\right)\;R_{z}\;\left(\gamma (t)\right),$$

which corresponds to the browser-side call rotation.set ($m_{y},m_{z},m_{x}$, "XYZ"). The same rotation is applied to both left and right virtual cameras.

In the implementation, head and waist IMU orientations were streamed via ROS 2 to the browser. We computed the yaw difference Δψ and applied the same head-only rotation to both the left and right virtual cameras, ensuring that whole-body turns (captured by the waist IMU) did not rotate the rendered viewpoint while head scanning was preserved.

### 2.3. Timing and Latency Measurement

All subsystems (capture, rendering, IMU I/O, and fusion) ran concurrently during latency measurements, so reported delays reflect the full pipeline rather than video projection alone.

## 3. User Study

This study used a within-subject 2 × 3 design (viewpoint height: head vs. waist; gap height: high/middle/low) to test the perceptual hypotheses stated in the Introduction. The primary dependent variables were (i) passability judgment, assessed by a 7-point confidence rating and the decision to attempt passage; (ii) locomotor behavior during passage attempts, quantified by the approach walking speed immediately before the gap; and (iii) post-condition subjective responses related to body image and spatial awareness.

### 3.1. Participants and Ethics

Nine healthy Japanese adults in their 20s (seven men, two women) participated. Eligibility required standing height within two standard deviations of the national average, normal ambulation, and no self-reported visual or balance impairments when using an HMD. All participants were naïve to the study purpose and provided written informed consent; they received small monetary compensation. The protocol was approved by the Internal Ethics Review Board of the Institute of Systems and Information Engineering, University of Tsukuba (Approval No. 2024R933, 14 November 2024 approval). Participants were recruited from 6 March 2025 to 9 March 2025. The study adhered to the Declaration of Helsinki. No identifiable information was collected; potentially identifying features in images were removed or obscured.

### 3.2. Experimental Procedure

**Figure 3:**
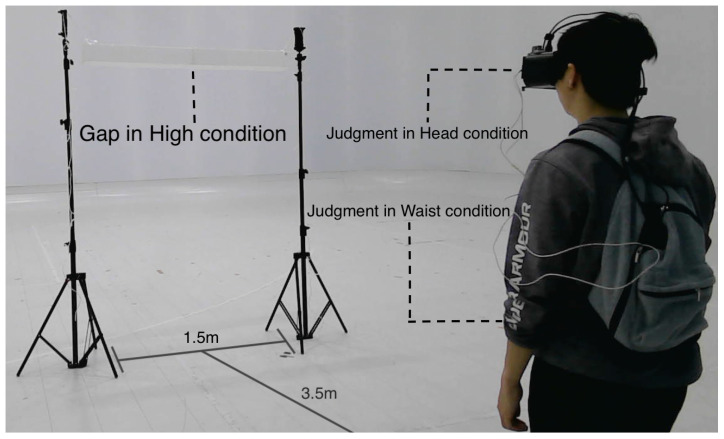
Making a judgment call on passing through a gap.

Before data collection, participants familiarized themselves with the system for several minutes. At each trial, we recorded (i) the confidence rating and pass/no-pass decision, (ii) whether a passage attempt was performed, and (iii) the approach walking speed immediately before the gap during passage attempts. Each trial began 3.5 m from an adjustable gap, as shown in Figure 3. Participants viewed the gap through the live feed and rated their confidence in passing on a seven-point scale (1 = no confidence, 7 = definitely passable). If the rating was ≥4, participants attempted to pass through the gap at a comfortable, self-selected speed; otherwise, the trial ended with a verbal response only. During passage, walking speed was measured over a 1–2 m segment immediately before the gap using an OptiTrack motion capture system, with markers on the backpack. System timestamps and motion capture time were logged on the same host for synchronization. Participants were instructed to avoid posture changes (e.g., squatting or sitting) and to stop if they felt unsafe. Viewpoint height and gap height were manipulated in a 2 × 3 within-subject design. The camera was positioned at either the participant’s eye level (head viewpoint) or waist level (waist viewpoint). In the waist viewpoint, IMUs were used to render head-only rotations in the displayed view. For yaw, we separated head rotation from whole-body turning by applying only the relative yaw (head minus waist). For the remaining axes (pitch and roll), head rotations were applied directly. In the head viewpoint, because the camera was mounted on the HMD, head rotations were already embedded in the incoming video stream; therefore, no additional IMU-based rotation was applied.

The adjustable gap was formed by a white horizontal bar, whose height from the floor was set to one of three levels based on each participant’s standing height: high (above the participant’s standing height, a height allowing the participant to consistently pass under the bar without collision), middle (approximately chest height), and low (below the waist). The gap width (distance between the two stands) was approximately 1.5 m, and the bar height was adjustable in 30 cm increments; therefore, the realized bar heights were discretized to the nearest achievable level. Each participant experienced all six combinations of viewpoint (head, waist) and gap height (high, middle, low) in a pseudo-randomized order with a fixed constraint at the middle gap: head–middle was always performed before waist–middle.

**Figure 4:**
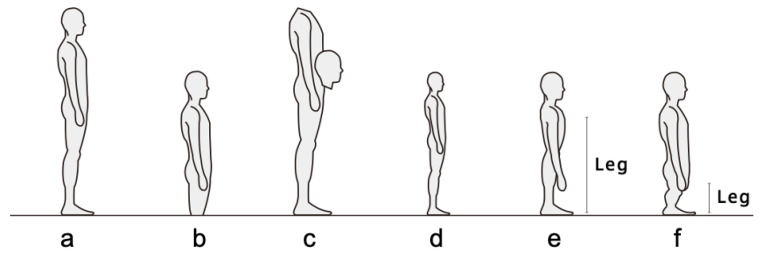
Body image questionnaire schematics: (**a**) usual; (**b**) grounded; (**c**) head-near-waist; (**d**) isotropic shrinkage; (**e**) vertical shrinkage; (**f**) leg shortening.

After completing six conditions, participants provided free-text feedback on comfort and spatial awareness and completed a body image questionnaire (Figure 4), selecting the option that best matched their experience. For the body image questionnaire, participants chose one schematic from six options: (a) usual body, indicating no perceived change in dimensions; (b) grounded look, in which overall dimensions are preserved but the body feels lower or more anchored to the ground; (c) head near the waist, depicting a lowered head position relative to the torso (repositioning only, segment lengths unchanged); (d) isotropic (proportional) shrinkage, where all linear dimensions are scaled by the same factor; (e) uniform vertical shrinkage, where the overall body height decreases while the relative proportions between the upper and lower body are preserved, and the torso thickness in the sagittal plane remains unchanged; and (f) disproportionate leg shortening, in which the legs appear shorter relative to the upper body compared with (e). To minimize ambiguity, (d) preserves the original aspect ratio, and (f) shortens only the leg segment.

### 3.3. Measurements and Analysis

We analyzed three outcomes: (1) passability confidence ratings, (2) body image questionnaire choices, and (3) walking speed (only in trials with attempted passage). Given n=9, we emphasize descriptive statistics and within-subject comparisons. Confidence ratings were compared between viewpoint conditions using the Wilcoxon signed-rank test across all gap heights. Walking speed was compared between viewpoint conditions using paired-sample *t*-tests (two-tailed). The significance level was set at α=0.05. All analyses were performed in Python 3.10.4 (pandas 2.2.3, numpy 1.26.4). Some scripts are provided in the Supplementary Materials; the remaining data and scripts are available from the corresponding author on reasonable request.

### 3.4. Statistical Notes

All statistical tests were two-tailed with α=0.05. We report effect sizes alongside *p*-values for the primary contrast of interest (head–middle vs. waist–middle), while other contrasts are reported with *p*-values to keep the main text concise.

For passability confidence ratings, we used Wilcoxon signed-rank tests for paired contrasts at each gap height. We emphasize the viewpoint-height effect at the middle gap height. For the head–middle vs. waist–middle comparison, we additionally report an effect size as $r=Z/\sqrt{N}$. For interpretability, we additionally report a standardized mean difference (*Cohen’s d_z_*) and a post hoc power estimate for the primary paired contrast, treating the 7-point ratings as quasi-interval; these are reported as descriptive indices only.

For walking speed, we treated speed as a continuous variable and exploratively compared them using paired *t*-tests (two-tailed; N=7 pairs for speed analyses) for comparisons between conditions. For paired *t*-tests, the effect size is reported as *Cohen’s d_z_*, computed as the mean of paired differences divided by the standard deviation of paired differences.

## 4. Results

### 4.1. System Latency

Prior to the user study, we quantified the end-to-end video delay for multiple resolution frame-rate settings with all subsystems active (including IMU processing), thus reflecting computational load beyond raw video projection. Delay was measured by imaging a digital stopwatch displayed on a PC monitor with the system’s stereo camera, while a third camera simultaneously captured both the source monitor and the HMD screen; latency (ms) was computed from the difference between the two time readouts. For each setting, six frames were sampled at ∼4 s intervals starting 1 min after system startup.

**Figure 5:**
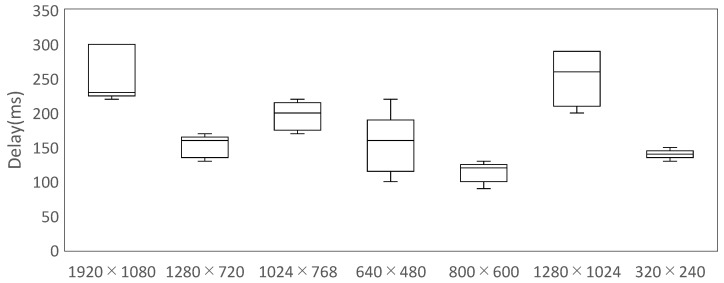
End-to-end latency across resolution frame-rate settings with all subsystems active; 800×600@60 fps remained <150 ms in all samples. Box plots show the median (center line).

Across the tested conditions (1920×1080@30 fps, 1280×720@60 fps, 1024×768@30 fps, 640×480@120 fps, 800×600@60 fps, 1280×1024@30 fps, 320×240@120 fps), the 800×600@60 fps mode consistently maintained latency below 150 ms with low variance, whereas higher resolution settings (e.g., 1280×1024, 1920×1080) exhibited larger means and variability, with several samples exceeding 150 ms. Based on this profile, 800×600@60 fps was selected for the user study; at this operating point, all recorded samples remained <150 ms with low variance (Figure 5).

### 4.2. User Study

#### 4.2.1. Passability Judgments

**Figure 6:**
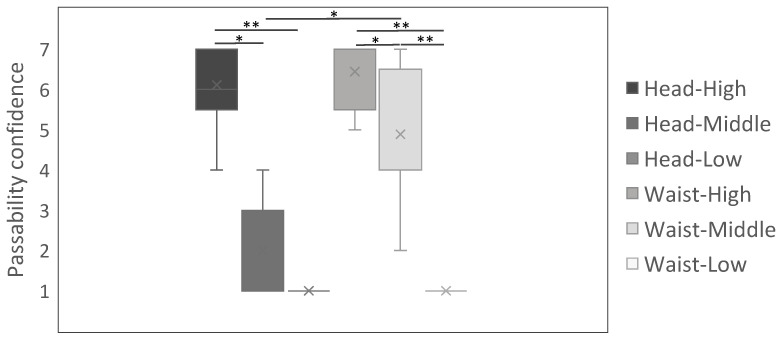
Judgment of passability across conditions (* *p* < 0.05, ** *p* < 0.01).

We first summarize the key pattern observed in passability judgments (Figure 6). The largest viewpoint-dependent change appeared for the borderline (middle) gap, where confidence was lower in head–middle than in waist–middle. Meanwhile, confidence was consistently high at the high gap and consistently low at the low gap across participants, regardless of viewpoint height.

Participants rated their confidence in passing through gaps on a 7-point scale under a 2 (viewpoint height: head vs. waist) × 3 (gap height: high/middle/low) design (Figure 6). Given n=9, Wilcoxon signed-rank tests were used for paired contrasts on the confidence ratings. Head–middle vs. waist–middle differed significantly (normal approximation z=2.5205; p=0.0129; r=0.84). For reference, when treating the 7-point ratings, the standardized mean difference was large ($d_{z}=1.38$); the corresponding post hoc power estimate for this contrast was 1−β=0.907.

**Figure 7:**
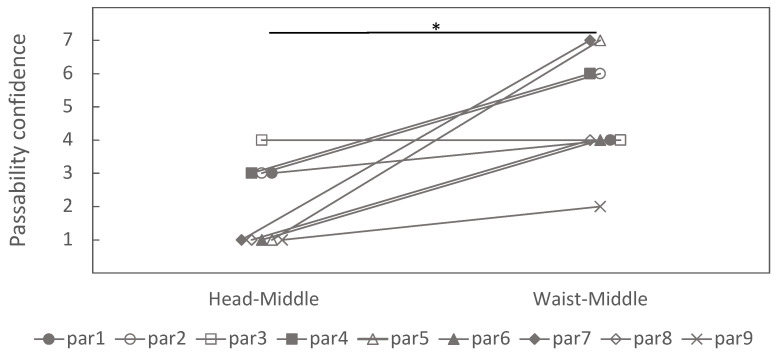
Judgment of passability in head–middle and waist–middle conditions (* *p* < 0.05).

Within the same viewpoint (head), head–high exceeded head–middle and head–low (p=0.0136 and p=0.008, respectively), whereas head–middle vs. head–low was not significant (p=0.52). With the viewpoint at the waist, waist–high exceeded waist–low and waist–middle exceeded waist–low (p=0.007 and p=0.008), and waist–high exceeded waist–middle (p=0.02). Holding gap height constant, head–high vs. waist–high and head–low vs. waist–low were not significant (both p=0.52), consistent with the observations that viewpoint-dependent changes were most evident at the middle gap. Eight of nine participants showed higher judgment values in waist–middle than head–middle (Figure 7).

#### 4.2.2. Body Image and Spatial Awareness Responses

**Figure 8:**
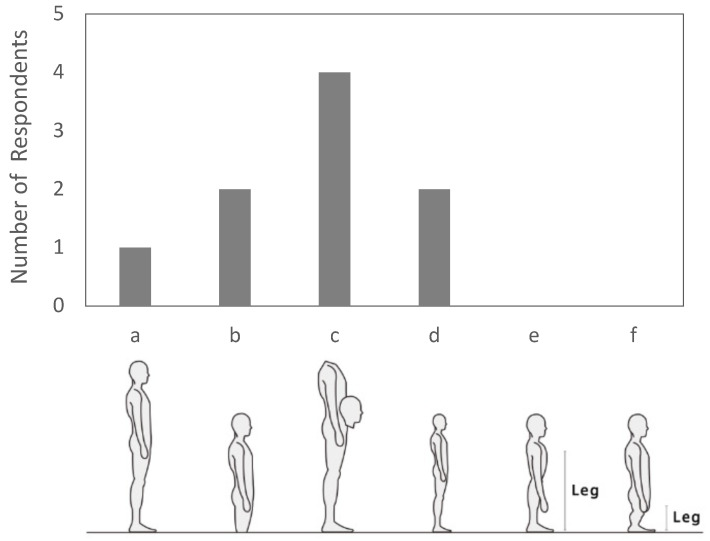
Selected body image: (**a**) usual; (**b**) grounded; (**c**) head-near-waist; (**d**) isotropic shrinkage; (**e**) vertical shrinkage; (**f**) leg shortening.

After completing all conditions, eight of nine participants selected a body image deviating from the baseline figure, with option (c) (head perceived near waist level) most frequent (Figure 4 and Figure 8). Free-text responses commonly included “felt smaller,” “head felt lower,” and “spatial scale changed.” Some participants also reported initial apprehension immediately after donning the device, citing the narrow field of view and unusual perspective. These responses are consistent with the shift in conscious body image and spatial awareness that accompanies viewpoint-height manipulation. Table 1 summarizes, for the waist–middle condition, how many participants endorsed each combination of passability judgment and body image option. One participant who selected the usual body image (a) reported a low passability judgment, whereas all other participants reported passability judgments of 4 or higher.

#### 4.2.3. Walking Speed

**Figure 9:**
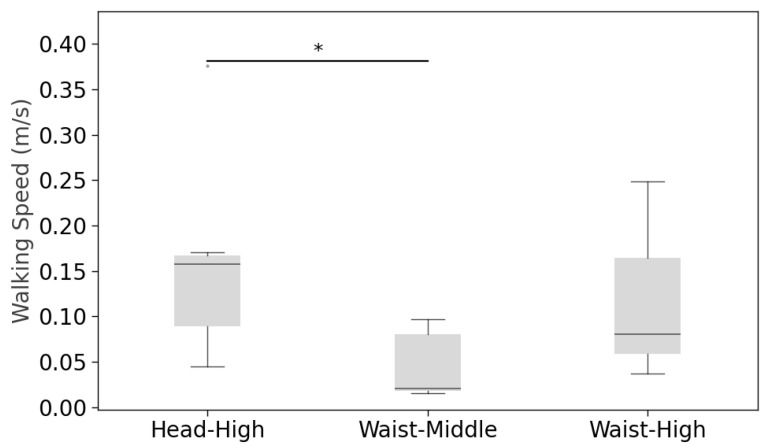
Walking speed during passing through attempts (* *p* < 0.05).

Walking speed (measured over the final 1–2 m before the gap) was analyzed only for trials where passability confidence ≥4 (Figure 9). Because one participant’s recordings were missing and another did not attempt the pass-through (passability confidence <4), the walking speed analysis included 7 participants. Within-subject paired two-tailed *t*-tests showed head–high > waist–middle (t(6)=2.8755, p=0.0379, 1−β=0.601, $Cohen’s\;d_{z}=1.00$); head–high vs. waist–high (t(6)=1.3086, p=0.3112, 1−β=0.154, $Cohen’s\;d_{z}=0.416$); and waist–middle vs. waist–high (t(6)=2.0635,p=0.0995, 1−β=0.378, $Cohen’s\;d_{z}=0.74$) were not significant. Descriptively, “high” gaps elicited slightly faster approaches, whereas waist–middle exhibited greater variability. The apparent difference between head–high and waist–middle should be interpreted cautiously, given the reduced sample size and the fact that speed was only observed for attempted passages (confidence ≥4), which censors low-confidence trials (especially at low gap heights).

## 5. Discussion

### 5.1. Answers to Research Questions

The key effect was confined to the middle (chest-level) gap, where passability confidence increased under the lowered viewpoint (waist–middle > head–middle).

Regarding the research questions, our discussion is summarized as follows:

- RQ1 (passability judgment): Viewpoint-height transformation affected passability judgments primarily at the chest-level (middle) gap. Lowering the viewpoint tended to increase judged passability, whereas clearly high or low gaps were largely insensitive to viewpoint.
- RQ2 (body representation): Self-reports were more consistent with a recalibration of body–environment scaling (altered perceived eye height) than with an explicit change in body morphology.
- RQ3 (walking speed): Changing viewpoint alone did not systematically alter walking speed at high gaps. In contrast, slowing emerged when approaching the middle gap under the lowered viewpoint, suggesting a dissociation between the passability judgment and cautious execution during approach.

### 5.2. System Implications

This prototype shows that natural, untethered walking with free mobility can be achieved with a lightweight, PC-driven pipeline: a waist-mounted fisheye stereo rig feeds a small on-body PC, which renders a virtual height view on a smartphone-shelled HMD (phone form factor shell with an embedded display, PC-driven). We stabilize the image by mapping fisheye frames onto a hemispherical mesh in Three.js. This warp regularizes peripheral distortion and preserves a coherent scene at modest computational cost, which in our indoor walking tests produced predictable behavior and stable perception without cloud offloading or desktop-class graphics. The design is intentionally pragmatic: resolution and field of view are limited, yet the assembly remained light, portable, and robust enough to support the within-subject experiment reported here.

Practically, two implications follow. First, eliminating the large desktop computer simplifies setup and improves repeatability during don/doff and corridor length walking. Video is rendered locally by the on-body PC to the embedded display in the smartphone-shelled HMD; a local server on the same PC hosts the browser-based warp, and no external network is used for video delivery. Second, hemispherical mapping provides sufficient stabilization for this use case: a lightweight browser-based warp preserved geometric plausibility without SLAM or heavy rendering.

Most importantly, even with standard resolution and field of view, the waist-mounted interface altered space-related judgments during real walking, suggesting that high-end optics or full-room tracking are not prerequisites for training and accessibility-oriented deployments. The present study foregrounds this perceptual impact under free mobility; as a practical next step toward deployment, we will examine longer sessions and varied lighting conditions.

### 5.3. User Study

As shown in Figure 6, the high gap height was associated with high passability judgment and the low gap height with low judgment, regardless of viewpoint height. For example, at head–high and waist–high, all participants reported confidence ≥4, whereas at head–low and waist–low, all reported 1. By contrast, at the middle (chest-level) gap, passability confidence depended on viewpoint height: it was higher in waist–middle and lower in head–middle. Thus, the middle gap height constitutes a borderline region (i.e., near the decision boundary) for passability. In everyday life, a lowered viewpoint typically co-occurs with a change in posture (e.g., squatting or sitting); in our manipulation, however, the perceived viewpoint was lowered while participants remained standing, making the experience unusual compared to everyday posture changes. Even so, pass-through judgments appeared anchored primarily to the currently seen viewpoint rather than to a preexisting body representation (i.e., an internal estimate of body size and eye height independent of the current visual input), suggesting that small changes in perceived scale have their greatest impact around this boundary, especially for the middle gap height.

Walking speed results, while underpowered for firm conclusions (speed analyses n=7), are consistent with a dissociation between a static decision made at the starting point and walking behavior during approach. When only the viewpoint changed, and the gap was high (head–high vs. waist–high), speed did not differ, implying that viewpoint change in itself did not slow locomotion. By contrast, under waist–middle, participants judged the gap passable yet approached more slowly in waist–middle than in head–high.

As summarized in Table 1, post-task body image selections bifurcated: several participants chose depictions of a smaller-seeming body (b or d; grounded or proportional shrinkage), and others chose a head-near-waist depiction (c). Selections of b/d were often accompanied by higher passability judgments, consistent with a visual scaling interpretation tied to the current viewpoint. Even so, we interpret this pattern as being more consistent with reliance on the currently seen viewpoint (visual scaling cues) than with a fixed, viewpoint-independent internal estimate. Importantly, choosing c did not imply low passability; at waist–middle, confidence remained high. Options implying leg-length change or other segment-specific alterations (e,f) were not endorsed, and one participant selected the usual body representation (a) with lower confidence at middle. Taken together, these patterns indicate a dissociation: the smaller self-depictions (b/d) align with the representation that guided pass-through judgments from the current viewpoint, whereas the head-near-waist depiction (c) can coexist with high confidence yet implies the upper torso traveling closer to the gap’s top edge. These selections index subjective internal representations and do not imply morphological change or guarantee objective pass-through feasibility.

The underlying mechanism remains uncertain, and the following explanation is speculative. One possible explanation for the selective slowdown at waist–middle is increased caution near the borderline region. One possibility is that participants reweighted visual scale cues near the borderline region, so caution emerged during approach even when the initial judgment was “passable.” This mechanistic account should be tested in future work by counterbalancing order and sampling multiple near-threshold gap heights to estimate psychometric functions.

### 5.4. Relation to Prior Work

Prior work shows that manipulating apparent body size or eye height shifts perceived scale and decision criteria in physically unchanged environments [18,19]. Ecological accounts formalize this as an eye-height ratio for passability judgments: when eye height is altered, the judged critical barrier height shifts while the ratio remains approximately constant [36]. Related work has also shown that manipulations of visual eye height shift perceived affordances, such as the judged critical aperture of a doorway-like opening [41]. Body-worn telepresence systems have also examined how camera placement and height affect remote observers’ experience for remote viewing [42]. Extending these ideas, we evaluate a first-person, freely mobile implementation and observe viewpoint-height effects concentrated at chest-level (middle) gaps, where small-scale changes most strongly impact the decision boundary.

Locomotion studies indicate that strategies for negotiating gaps scale with body dimensions (e.g., shoulder width) [37]. In our data, viewpoint-height manipulation shifted confidence only near the boundary and produced selective slowing at waist–middle, while speed at high gaps was unchanged. Limited endorsement of proportional shrinkage and the absence of leg-length changes, together with frequent head-near-waist selections, are consistent with a visual scaling account in which the current viewpoint repositions the decision boundary; our data do not provide evidence for morphological reparameterization. Related augmented reality (AR) and virtual reality (VR) studies have reported experiential or social distance shifts under raised or lowered viewpoints [25,26,27,30,31]; here, we extend such experiential shifts to concrete passability judgments during natural locomotion with head–body contingencies preserved, and we show that cautious approach emerges specifically where the decision boundary is repositioned by the viewpoint change.

### 5.5. Applications to Training and Accessibility

Our results align with a broader trend in which wearable devices are used to shape physical activity and everyday behavior. Umbrella reviews report that activity trackers and similar wearables can increase physical activity and reduce sedentary behavior in adults [43]. Low-cost VR-based learning systems have also been explored for procedural training across diverse age groups [44]. Our findings indicate two practical use cases:

- Child-height perspective training. Lowering the viewpoint allows caregivers and parents to experience child-level spatial scale. High shelves and objects on desks become harder to reach, and adults may appear larger and more imposing, informing room layout and day-to-day safety practices.
- Wheelchair-level perspective. Presenting a seated viewpoint helps clinicians and designers judge how easily doors and corridors can be passed through and communicate body–environment scaling during posture transitions. This includes evaluating standing-capable mobility solutions (e.g., Qolo [45]), where perceived viewpoint-height changes as users switch between seated and upright modes.

For targeted evaluations, we recommend measuring changes in gap passability judgment, walking speed, and route choice in realistic mock-ups, as well as decision-making; when posture transitions are involved, include pre/post-comparisons across seated and upright modes.

### 5.6. Limitations

Several limitations should be noted. First, the sample size (n=9) limits generalizability and reduces the robustness of inferential testing. Participants were young, healthy adults from a single context, with a male-skewed sample and limited balance in gender, age, and cultural diversity. Future studies should recruit larger and more diverse cohorts and analyze the data with mixed-effects models to better account for subject-level variability. In addition, because head–middle always preceded waist–middle, sequence effects cannot be fully excluded; counterbalancing the order (e.g., Latin-square blocking) would strengthen causal interpretation.

Second, our walking-speed analyses were conditioned on attempted passages (confidence ≥4), which censors low-confidence judgments and may bias the behavioral sample. Moreover, the analyzable sample for speed was reduced (n = 7), so null effects may reflect limited statistical power. A more comprehensive design would include multiple near-threshold gap heights and estimate psychometric functions for passability criteria rather than relying on discretized middle/low settings that were not calibrated to a specific threshold.

Third, we transformed viewpoint height while holding other visual parameters constant. Improvements in optics and sensors may change the subjective experience (e.g., comfort) and potentially the strength of the observed effects. Body representation was assessed primarily via conscious self-reports; adding implicit indices (e.g., shoulder rotation at gaps) would help test whether body representation changes occurred. Furthermore, the schematic body image questionnaire is study-specific and has not been psychometrically validated, so the reliability with which participants distinguish the categories remains uncertain. Finally, we recorded only a few latency samples per mode, so short-term jitter may be underrepresented. In future work, we will sample latency more densely and report standardized effect sizes with confidence intervals, while controlling for multiple comparisons.

## 6. Conclusions

In this study, we developed a waist-mounted interface for mobile viewpoint-height transformation by combining a waist-mounted fisheye camera with a smartphone HMD case. The primary contribution is demonstrating that this lightweight, on-body configuration can reliably shift spatial passability judgments during real walking, without relying on high-end optics or external tracking. Lowering the viewpoint to waist height affected decision-making, and in a subset of conditions, approach behavior (walking speed) also changed. In particular, a waist-level viewpoint increased passability judgment, most clearly for the borderline (middle) gap height.

Body image reports changed but did not align with these judgments and rarely endorsed proportional shrinkage or leg-length change, pointing to an update of visually driven decision criteria tied to viewpoint height rather than morphological change. Behavioral and subjective measures were not always concordant. These results support the idea that viewpoint height serves as a criterion for determining the scale relationship between the body and the environment in real-world judgment tasks, and they inform training scenarios (e.g., caregivers/parents experiencing the child’s viewpoint) and accessibility-oriented design.

This study is exploratory and has important limitations, including a small sample size, incomplete counterbalancing with potential sequence effects (e.g., fixed ordering at the middle gap), and task-specific constraints (e.g., walking speed analyses only for attempted passages). As next steps, we will increase the sample size and fully counterbalance conditions to confirm robustness and assess generalizability across tasks and settings.

## Figures and Tables

**Table 1:** Counts by passability judgment (rows) and body image (columns).

		Body Image					
		**a**	**b**	**c**	**d**	**e**	**f**
Passability judgment	1	0	0	0	0	0	0
	2	1	0	0	0	0	0
	3	0	0	0	0	0	0
	4	0	1	2	1	0	0
	5	0	0	0	0	0	0
	6	0	0	1	1	0	0
	7	0	1	1	0	0	0

## Data Availability

The minimal browser-based rendering code is provided in the Supplementary Materials. The remaining data and analysis scripts are available from the corresponding author upon reasonable request and are not publicly available due to privacy and ethical restrictions.

## Supplementary Materials

The following supporting information can be downloaded at: https://www.mdpi.com/article/10.3390/s26020372/s1, Table S1: Raw data (Excel spreadsheet, .xlsx); File S1: sample.html (minimal implementation for the fisheye SBS rendering).

## Abbreviations

The following abbreviations are used in this manuscript:

XR	Extended Reality
VR	Virtual Reality
AR	Augmented Reality
HMD	Head-Mounted Display
IMU	Inertial Measurement Unit
ROS 2	Robot Operating System 2
SBS	Side-by-Side (stereo presentation)
FOV	Field of View
ms	Milliseconds
fps	Frames Per Second
