# Supplementary material for: A Waist-Mounted Interface for Mobile Viewpoint-Height Transformation Affecting Spatial Perception
Source: Sensors (Basel). 2026 Jan 6;26(2):372. doi: 10.3390/s26020372 (PMC12845937; doi:10.3390/s26020372)
Supplement: Supplementary file 1 [file sensors-26-00372-s001.zip › File S1 sample.html]

Stereo Fisheye (SBS)


Initializing Cameras...
